# The Effects of Phonological Complexity on Word Production in French-Speaking Children

**DOI:** 10.1177/00238309241237473

**Published:** 2024-04-02

**Authors:** Margaret Kehoe

**Affiliations:** Université de Genève, Switzerland

**Keywords:** Phonological complexity, phonological production, French phonological development, word complexity measures

## Abstract

Word complexity indices, such as the Index of Phonetic Complexity (IPC) and the Word Complexity Measure (WCM), code a word in terms of featural and structural properties that pose difficulty in phonological development. Studies have investigated the influence of complexity indices on vocabulary development; however, few have examined their influence on consonant accuracy. Furthermore, these indices were developed for English-speaking children and have not been widely applied to other languages. This study investigates whether a word’s phonological complexity influences how accurately it is produced in French-speaking children. Four databases consisting of the productions of children (*n* = 74), aged 1;11 to 4;9, were analyzed. Words were coded in terms of the IPC, WCM, and parameters that add complexity during phonological development. Using mixed-effects logistic regression, we examined whether phonological complexity as determined by the IPC, WCM, or by alternative indices better accounts for the influence of complexity on production. We also investigated whether the accuracy of a target sound/structure was influenced by a word’s complexity. Results indicated that complexity based on the IPC or WCM significantly influenced consonant accuracy; however, indices tapping fewer features provided superior model fit. At younger ages, the presence of fricatives/liquids and, at all ages, the presence of alveopalatal fricatives, codas, and clusters significantly influenced accuracy. Findings were inconclusive as to whether whole word complexity influenced the accuracy of a target sound/structure. Results suggest that current complexity indices provide only approximate indications of how featural and structural properties of words influence production.

## 1 Introduction

Linguists have long been intrigued by the notion of phonological complexity. They have attempted to quantify it by comparing the size of consonant and vowel inventories, the markedness of consonants, the complexity of syllable structure, and the transparency of phonological alternations in languages of the world (Lindblom & Maddieson, 1988; Maddieson, 2005; Shosted, 2006). Child language researchers have also been interested in phonological complexity and how it influences child speech. Some have considered phonological complexity when developing phonological tests (de Almeida et al., 2019; dos Santos & Ferré, 2018) or when making decisions about which sounds to target in phonological intervention (Gierut, 2001, 2007). Others have developed indices of word complexity by coding words in terms of featural and structural properties considered to be phonetically or phonologically complex (Jakielski, 2000, 2002; Stoel-Gammon, 2010). These indices have been employed in various ways, for example, to examine whether a word’s phonological complexity influences vocabulary development (Kehoe et al., 2018, 2020; Kern & dos Santos, 2017) or whether it increases the likelihood of a word being stuttered (Coalson et al., 2012; Howell et al., 2006).

In this study, we ask a more basic question “Are phonologically complex words more difficult to produce accurately?” We first investigate the influence of two known word complexity indices, the Index of Phonetic Complexity (IPC, Jakielski, 2000) and the Word Complexity Measure (WCM; Stoel-Gammon, 2010), on consonant accuracy. We then examine whether alternative indices containing fewer or different features better capture the influence of complexity on word production. Although it is logical to assume that phonologically complex words will be produced less well than less complex words, few studies have examined the influence of word complexity on the accuracy of children’s productions. We also examine whether the effects of complexity change across age by analyzing data from younger (2 years) and older children (3–4 years).

Thus, this study aims to validate existing word complexity measures and compare them with alternative measures. Such validation is important for theoretical and practical reasons. Linguistic theories offer guidelines on which phonological properties in a given target word are more or less complex (see next section). However, they do not inform us on whether the presence of these features in a target word will influence production accuracy and which features when combined influence production accuracy the most. This study intends to respond to these questions. From an applied perspective, a valid word complexity measure has many uses. As hinted above, it may be employed to select word stimuli when developing speech and language tests and it may guide the selection of words in speech and language intervention. In both of these applications, it is important to control phonological complexity so that it does not interfere with the target feature of interest. Phonological complexity may also be directly manipulated to examine its effects on vocabulary and fluency development (Howell et al., 2006; Kehoe et al., 2018, 2020). However, all of these applications rely on having a valid measure of word complexity, something which is not yet assured by the existing research. In addition, much of the research has concentrated on English-speaking children and it is important to determine whether the complexity parameters selected for English are equally useful for other languages. The effects of complexity parameters may vary depending on the frequency and distribution of linguistic features or structures in the target language. In the following paragraphs, we define phonological complexity and consider studies that have examined its effect on children’s speech.

### 1.1 Phonological complexity: a definition

Complexity refers to the number of descriptor elements needed to define a system: the more the descriptor elements, the more complex the system (Gierut, 2007). It is often defined in terms of structural considerations. A phonetic/phonological property with more elements and structure is more complex than a phonetic/phonological property with fewer elements and less structure. Structural considerations can explain why syllables containing clusters or codas are denoted as more complex than syllables without these elements. Similarly, complexity as based on cardinality, the number of elements, may explain why words containing three or more syllables are more complex than those of one or two syllables.

In terms of segmental complexity, Lindblom and Maddieson (1988) classified obstruents and sonorants according to three groups based on articulatory difficulty. Their system separated consonants with basic from those with elaborated articulations, the latter referring to sounds produced with nondefault modes of voicing (e.g., breathy or creaky voice) and nasality (e.g., prenasalization, nasal release), or which had supplementary manner (e.g., ejectives, implosives, clicks) or place features (e.g., alveopalatal, retroflex, uvular). Set 1 had basic consonants, Set 2 had elaborated consonants, and Set 3 had a combination of elaborated features as suggested by the following: /k/ (Basic), /kʷ/ (Elaborated), and /kʷʼ/ (Complex), whereby / kʷʼ/ indicates a labialized velar ejective.

Important to note is that alveopalatal and uvular consonants were considered to have nonbasic articulations. Voiced fricatives were also considered more elaborated than voiced stops because they involve nonspontaneous voicing (fricatives favor voicelessness) and the control of the noise source. The structural and featural complexity used to describe adult systems also receives support in complexity indices designed for child speech.

### 1.2 Indices of complexity in child speech

Jakielski (2000, 2002, 2022) developed a measure of word complexity (the IPC) based on eight parameters known to pose difficulty for children in production. Stoel-Gammon (2010) created a similar type of measure (WCM), which assesses phonological elements (word patterns, syllable structures, and sound classes) that do not occur in the speech of typically developing two-year-olds. Although Jakielski’s (2000) index purports to measure articulatory (phonetic) complexity and Stoel-Gammon’s (2010) measures phonological parameters, the indices share similar complexity features and are based on phonological developmental findings. For simplicity’s sake, we refer to both measures as indices of phonological complexity. They are point-based measures, in which the target word receives a point or a score if the complexity parameter is present.

Both indices pay attention to the featural properties of sound classes. In terms of place of articulation (PoA), they provide points to words containing dorsals (not labials and coronals), and in terms of manner of articulation (MoA), they assign points to words containing fricatives, affricates, and liquids (not stops, nasals, and glides). Words receive multiple points depending on the number of dorsals, fricatives, affricates, and liquids they contain. Both indices pay attention to complex syllable structure. Words containing clusters (consonant sequences) and word-final consonants are assigned complexity points. Similarly, both systems assign points for “long” words (i.e., words containing three or more syllables). In terms of differences, the WCM assigns an additional point for words containing voiced fricatives and affricates. It also considers the stress pattern of words. Words containing stress on any syllable but the first will receive a complexity point. In contrast, the IPC provides complexity points for place variegation. Both singleton consonants and clusters are awarded points when PoA varies from one singleton consonant to the next (*gâteau* /gato/ “cake” versus *lune* /lyn/ “moon,” whereby only *gâteau* has PoA variegation) or when there is PoA variegation within a cluster (e.g., *ski* /ski/ “ski” vs. *stop* /stɔp/ “stop,” whereby only *ski* has PoA variegation in the cluster). Finally, the IPC assigns points for rhotic vowels, whereas vowels are not taken into consideration in the WCM. The current study focuses on French-speaking children and the parameters that refer to noninitial stress and rhotic vowels are not relevant to the French language. The different complexity features assessed in the two systems are presented in Table 1.

**Table 1.** Comparison of complexity parameters in the index of phonetic complexity (IPC, Jakielski, 2002) and the word complexity measure (WCM, Stoel-Gammon, 2010).

Complexity parameter	Point assigned for:	Jakielski (2002)	Stoel-Gammon (2010)
		IPC	WCM
Place of articulation	Dorsals	✔	✔
Mode of articulation	Fricatives^a^/	✔	✔
	Liquids		✔
	Voiced fricatives^a^		✔
Syllable structure	Word-final consonants	✔	✔
	Clusters	✔	✔
Word length in syllables	Words of 3 + syllables	✔	✔
Place variegation	Singletons	✔	
	Clusters	✔	
Vowels	Rhotic vowels	✔	
Stress pattern	Words without initial stress		✔

a These complexity parameters also include affricates. However, they are not considered because French does not have affricates in its phonemic inventory.

Some authors have criticized the IPC, arguing that several of its parameters (e.g., word-final codas and place variegation) are already mastered in early speech development (Ratner, 2005). The WCM is considered to be a more appropriate measure for preschool children because it includes later acquired constructs such as voiced fricatives and affricates and also includes a word stress parameter (Coalson et al., 2012). We also ask whether there are other complexity parameters not included in the IPC and WCM that should be considered. In adapting the IPC to Arabic and Berber, Gayraud et al. (2018, see below) added a parameter to account for the contrast between simple versus complex articulations, the latter involving secondary articulations such as pharyngealization and labialization. In adapting the WCM to Swedish, Marklund et al. (2018) added trills as a complexity parameter because they are late-acquired in Swedish and several other languages (Stemberger & Bernhardt, 2018). In the case of French, the language under investigation, alveopalatal fricatives (ʃ, ʒ) are known to pose difficulties in acquisition (Aicart-de Falco & Vion, 1987; Kehoe & Philippart de Foy, 2023). MacLeod et al. (2011) identified them as late-acquired in a normative study of speech sound acquisition in Canadian French children. They are also among the latest sounds acquired in 27 different languages according to a cross-linguistic review of speech sound acquisition by McLeod and Crowe (2018). Word-final voiced obstruents have also been identified as complex elements in speech production (Bernhardt & Stemberger, 1998). They pose an aerodynamic challenge because children need to obtain the right balance of intraoral and subglottal pressure to maintain voicing at the end of a word (Smith, 1979). Glaspey et al. (2022) found them not to be fully acquired until late in acquisition in the connected speech of English-speaking children. Kehoe (2021) found voiced obstruents to be the codas the least well produced by French-speaking children, aged 3 to 6 years. Thus, the presence of alveopalatal fricatives and final voiced obstruents are additional parameters to consider when examining complexity effects on word production in French.

### 1.3 Studies investigating complexity in child speech

Gayraud et al. (2018) employed an adapted version of the IPC to examine the development of phonological complexity across four different languages: Arabic, Berber, English, and French. They selected 50 words from four different samples: an adult dictionary of each language, child-directed speech, target words produced by four children (first 50 words), and the children’s actual productions. The purpose of the study was to compare complexity across the four different languages, and, indeed, the authors found evidence of cross-linguistic differences: Arabic was characterized by higher degrees of complexity than the other three languages, and children acquiring Arabic produced more complex words than children acquiring Berber, English, or French. The authors also found that the IPC parameters contributed differently to overall complexity depending on the language. Manner and place of articulation contributed significantly to complexity in all languages whereas other parameters (e.g., clusters, word-final consonants) had differential effects depending on the language. Important for the purposes of the current study was the finding that the IPC parameters were not equally difficult for children to produce. Some parameters (e.g., dorsals, word length) appeared to be overcome more easily than others (e.g., clusters).

Although not employing the word complexity indices of Jakielski (2000) and Stoel-Gammon (2010), several authors have examined the influence of isolated complexity features on word production. Warnier et al. (2023) investigated the influence of word length (number of syllables) and syllable structure (singleton vs. consonant clusters) on two measures of word production: percent consonants correct (PCC) and whole word proximity (WWP; Ingram, 2002) in French-speaking children. The WWP is a measure that takes into account the number of segments (consonants and vowels) and correct consonants in a production. Three-year-old French-speaking children produced words varying from one to four syllables and which contained tautosyllabic clusters (i.e., clusters within a syllable). The authors documented significant main effects of word length and syllable complexity on word production. Words containing tautosyllabic clusters were characterized by lower PCCs than words containing singleton consonants, and the number of syllables in the word influenced the PCC. Interestingly, performance on words containing two to three syllables was higher than on words containing one and four syllables. Whereas it is understandable that four-syllable words may be characterized by lower accuracy scores than two- and three-syllable words, it is less understandable why one-syllable words should have lower accuracy scores. The authors argued that the poor results on one-syllable words were due to the fact that they contained more complexity features (e.g., more clusters) than the di-syllabic and tri-syllabic words.

In a later analysis, Warnier et al. (2023) examined whether combining complexity points influenced word production measures. They selected 12 words that combined at least two of the following complexity points (presence of clusters, word-internal or -final codas, and word length of four syllables). Words combining several complexity points had lower WWP scores than words containing none or only one complexity point, although this finding was not replicated for the PCC results. Focusing on word length (words of one- to three-syllables), Berube et al. (2020) did not find that word length influenced consonant accuracy in typically developing French-speaking children, aged two to four years, although it did influence the production of certain types of consonants (e.g., liquids) in phonologically disordered children.

Using nonwords, dos Santos and Ferré (2018) observed a decline in word repetition scores in three-syllable versus two-syllable words and in words containing two clusters versus one cluster. They tested monolingual and bilingual French-speaking children, aged 5 to 8 years, with and without developmental language delay (DLD). The effects of complexity were observed across all children but emerged as significant predominantly in children with DLD.

### 1.4 Factors apart from complexity that influence consonant accuracy

Apart from phonological complexity, other factors may influence a word’s production accuracy. Sosa and Stoel-Gammon (2012) examined factors influencing English-speaking toddlers’ (15 children aged between 2;0 and 2;5) word accuracy when using WWP as output measure. Target words were elicited during a free play session. They calculated phonological complexity by averaging the age of acquisition (AoA) for each consonant in the target word, employing norms established by Smit et al. (1990). The authors found that phonological complexity and phonological neighborhood density (PND) influenced WWP scores. Words with earlier acquired consonants and with denser neighborhoods were produced more accurately than words with later acquired consonants and less dense neighborhoods. Word frequency and phonological probability (i.e., the relative frequency of individual sounds and sound sequences) did not contribute significantly to regression models. Other studies have shown strong correlations between vocabulary size and phonological performance (Fletcher et al., 2004; Kehoe et al., 2015; Paul & Jennings, 1992). In the current study, along with phonological complexity, we will include PND and vocabulary knowledge as potential factors influencing word production.

### 1.5 Current study

In sum, little research has examined whether complex words as determined by the IPC or WCM are characterized by lower production accuracy than less complex words. This study aims to validate these measures and compare them to alternative measures of word complexity. The few studies that have investigated the influence of complexity indices on word production have focused on very young children (Stoel-Gammon, 2010). Thus, another aim is to broaden the empirical base to include children through to four years. We also test the validity of the IPC and WCM by applying them to French acquisition data, to determine whether the complexity parameters developed for English may be generalized to other languages.

Specifically, this study investigates the influence of a word’s phonological complexity on the accuracy of children’s word production. Words will be coded in terms of the complexity parameters of the IPC and WCM and parameters that stem from findings in phonological developmental research (e.g., presence of alveopalatal fricatives, final voiced obstruents). We use four different databases of French-speaking children’s word productions spanning the age range from 1;11 to 4;9 years. The original databases included monolingual and bilingual children; however, we analyze the results of monolingual children only to avoid any confounding effects of cross-linguistic interaction. The reason for using four datasets is to abstract away from isolated effects that may arise due to the selection of individual words in a single database. It also allows us to examine whether the influence of complexity changes with increasing age. Because the complexity parameters refer mainly to consonants, we employ PCC as the dependent variable.

First, on the basis of correlational analyses, we examine whether a word’s phonological complexity (based on the IPC and WCM) is negatively correlated with its overall production accuracy. Second, using mixed-effects logistic regression, we examine whether the IPC and the WCM significantly account for an individual word’s consonant accuracy, or whether the combination of fewer parameters and/or the inclusion of other factors better account for model fit. In these analyses, we control for age, gender, and SES. Consonant accuracy improves with increasing age (Smit et al., 1990), and some studies have documented gender and SES effects on consonant production: girls make fewer speech errors than boys (Kenney & Prather, 1986), and children from higher SES backgrounds have better articulation abilities than children from lower SES backgrounds (Campbell et al., 2003). As mentioned above, we also include other factors known to influence word accuracy such as PND and vocabulary knowledge.

Finally, we conduct additional analyses on children’s productions to further study complexity effects on production accuracy. We examine children’s outputs to confirm that the complexity parameters of the target form are indeed associated with poor accuracy of the same parameter in the output form. We also examine whether word complexity influences the production accuracy of a specific target sound or structure. Whereas in the main analyses, we examine whether word complexity influences how accurately the whole word is produced (PCC for the entire word), in the additional analyses, we examine whether word complexity influences how accurately a single target sound/structure is produced. The second set of analyses should help to address whether the influence of complexity is additive (sum of individual complexity components) or interactive/cumulative (more than the sum of individual complexity components). For example, we study if a cluster is more accurate when situated in a phonologically simple (e.g., *train* /tʁɛ̃/ “train”) than in a complex word (e.g., *tracteur* /tʁaktœːʁ/ “tractor”). If complexity effects are due to the complexity of the individual components, the accuracy of the target sound/structure should be similar whether it is situated in a more or less complex word. If complexity effects are due to the interacting or accumulated effects of different structures/features in a word, the accuracy of the target sound/structure should be less in a more complex than in a less complex word. Several authors have studied such effects when complexity has been defined in terms of the presence or absence of word stress or by the length of the word (Bérubé et al., 2020; Brosseau-Lapré & Rvachew, 2014); however, few authors have examined these effects when complexity is defined in terms of the featural and structural parameters of word complexity measures.

## 2 Method

### 2.1 Database

The study contains four datasets of monolingual French-speaking children’s word productions; they are summarized in Table 2. Each dataset contains approximately 20 children (range: 15 to 21). The children produced at least 25 different stimulus words (range: 25 to 85) and words varied in complexity from 0 to 9 based on the IPC and from 0 to 7 based on the WCM. The youngest children (Kehoe et al., 2023; Kehoe & Havy, 2019) produced only one- and two-syllable words, and thus, the parameter of word length was not tested in these datasets.

**Table 2.** Summary of the four datasets of French-speaking children’s word productions.

Datasets	Age	No. of children	No. of words	IPCrange	WCM range	No. of syllables	No. of items
Kehoe and Havy (2019)	2;6	17	29	2-6	1-6	1-2	755
Kehoe et al. (2023)	1;11-3;1	21	35	0-7	0-6	1-2	695
Kehoe and Cretton (2021)	2;7-4;3	21	25	2-9	1-7	1-4	1544
Kehoe and Girardier (2020)	3;0-4;9	15	85	0-8	0-7	1-4	1797

The data were collected under similar methodological conditions. Children took part in an object or picture naming task in which they were encouraged to produce multiple productions of the stimulus words.^1^ In Kehoe and Havy (2019), the children were tested at the speech laboratory at the University of Geneva, and, in Kehoe et al. (2023), the children were tested in a quiet room in their homes. In the other studies, the children were tested in quiet rooms in their schools or crèches/kindergartens. Apart from the stimulus words selected for the different studies, words spontaneously produced by children (e.g., children said *framboise* “raspberry” instead of *fraise* “strawberry”) were included in the database with the condition that all words were produced at least 10 times in total across several children within a given dataset (i.e., each word was represented at least 10 times within a dataset).

In the younger children, vocabulary knowledge was determined by asking parents to complete the Mots et Phrases form of l’Inventaire Français du Développement Communicatif (IFDC) (Kern & Gayraud, 2010) (the French adaptation of the MCDI). It consists of a list of 690 words organized into 22 semantic categories. Parents were asked to indicate whether their child produced the word. The IFDC is sensitive to vocabulary development over time and has strong short-term test–retest reliability (*r* = .90; Kern & Gayraud, 2010). In the older children, expressive vocabulary was tested using the subtest “Dénomination Phonologie/Lexique” of the test battery “Evaluation du développement du langage oral chez l’enfant de 2 ans 3 mois à 6 ans 3 mois” (EVALO2-6; Coquet et al., 2009). Children were required to name a series of 40 items (32 nouns and eight verbs). If the children spontaneously named the picture, they received a score of 2. The raw rather than the standardized score was included in the statistical models.

Data were recorded with unidirectional condenser microphones and portable digital recorders placed on a table approximately 30–40 cm from the child’s mouth. The recordings were segmented and transcribed using Phon, a software program for the analysis of phonological data (Rose et al., 2006). Native French-speaking graduate students completed the transcriptions. They coded the children’s productions in broad phonetic transcription. When reviewers were in doubt about the transcription (e.g., [ʃ vs. s]), they were advised to listen to the production a maximum of three times and make a decision about which sound the child had produced. Sound distortions (e.g., interdental or lateralized [s]) were not taken into consideration. The transcribed data were transferred to Excel and coded according to the phonological criteria under consideration. Calculations of PCC were computed automatically for each word in Phon. To provide an example, cheval /ʃəval/ “horse” produced as [ʃova] was coded as 2/3 for PCC. Inter-rater reliability for the phonetic transcription was conducted in each study and, in all cases, it exceeded 80% point-to-point reliability.

### 2.2 Data coding

The target words across all the datasets were coded in terms of their phonological complexity and PND. First, we coded each word in terms of the parameters of the IPC. That is, a point was assigned to each word if it contained a dorsal consonant (e.g., *camion* [kamjɔ̃] “truck”),^2^ a fricative or liquid (e.g., *avion* [avjɔ̃] “plane”; *balle* [bal] “ball”), a final consonant (e.g., *balle* [bal] “ball”), three or more syllables (e.g., *animal* [animal] “animal”), two or more singleton consonants with different PoAs (e.g., *balle* [bal] “ball,” which has labial and coronal PoAs), a cluster, either tautosyllabic (i.e., a cluster that occurs within a syllable; e.g., *crayon* [kʁejɔ̃] “pencil”), or heterosyllabic (i.e., a cluster that is split across two syllables; e.g., *tracteur* [tʁaktoeːʁ] “tractor”), and a cluster whose individual elements varied in PoA (e.g., *plume* [plym] “feather,” which has both labial and coronal segments in the cluster /pl/). Second, we coded each word in terms of the parameters of the WCM. Thus, we coded each word in terms of the presence of dorsal consonants, fricatives, liquids, final consonants, three or more syllables, clusters (tautosyllabic only),^3^ and voiced fricatives (see Note 2) (e.g., *avion* [avjɔ̃] “plane”). Third, we coded words in terms of two additional parameters: the presence of alveopalatal fricatives (e.g., *vache* [vaʃ] “cow”) and word-final obstruents (e.g., *robe* [ʁɔb] “dress”). The complexity parameters for the individual target words in each database can be found in OSF https://osf.io/fh8ry/. Finally, all target words were coded for PND using the values generated by the Lexique3 database, a corpus of adult language (New et al., 2007).

### 2.3 Statistical analyses

Data were analyzed using mixed effects logistic regression, which allowed us to model production accuracy on the basis of binomial data. The analyses were performed using R statistical software (R Development Core Team, 2020) and the lme4 package (Bates et al., 2015) for mixed-effects models. The dependent variable was a proportion score: the number of consonants correct over the total number of consonants for each word production. We also included a “weights” argument in the model set to the number of total consonants to take into account that a proportion (e.g., 0.5) could refer to different numerators and denominators (e.g., 1/2, 2/4, 3/6, etc.).

The analyses included the control variables, age (in months), gender, and socioeconomic status (SES, based on the number of years of education of the mother) and the predictor variables, phonological complexity, PND, and the child’s vocabulary knowledge (Vocab), the latter based on raw scores of the French MCDI or the vocabulary test, EVALO2-6. To test the influence of phonological complexity, we proceeded in several steps. We first built an empty model with minimum random variables (random intercepts), then added control variables, predictor variables, and their interactions, and then eventually added a more complex random structure (random slopes). At each step, we removed variables that were not significant. For each dataset, we first fitted a model in which phonological complexity was the IPC score based on all eight parameters of the IPC (minus rhotic vowels). We then fitted a second model in which phonological complexity was the WCM score based on all eight parameters of the WCM (minus initial unstress). Following this, we fitted a third model in which phonological complexity was based on what we refer to as the Custom index, an index that was individually determined for each dataset. To calculate this index, we entered the 10 individual phonological complexity parameters: the eight parameters of the IPC and WCM and two additional parameters (e.g., alveopalatal fricatives, final voiced obstruents) into our model. Each parameter was coded in terms of 0 (not present) or 1 (present), or in terms of the number of segments that characterized the target parameter (e.g., number of dorsals, fricatives, liquids, clusters, etc.). We removed individual parameters that were not significant one by one (based on their z values) until only significant (or marginally significant) parameters remained in the model. We then calculated a new complexity index (i.e., Custom index) based on the parameters that were significant (or marginally significant). It was this index that was used in the third model. We compared the first (based on the IPC), second (based on the WCM), and third (based on the Custom index) models to determine which was the best model according to Akaike information criterion (AIC).^4^ This procedure was conducted for all datasets. The random part of the model included random intercepts for participants and items (i.e., words). We also tested whether by-participant random slopes for SES, age, and vocabulary and by-item random slopes for phonological complexity and ND improved model fit. The inclusion of random slopes did not improve model fit or resulted in overfitting (singular fit) in all analyses except for Dataset 4 (Kehoe & Girardier, 2020) in which a by-item slope for phonological complexity improved model fit for model 1, the analysis in which phonological complexity was based on the IPC. All models were fitted using maximum likelihood estimation.

## 3 Results

### 3.1 Correlational analyses

Table 3 presents the correlation coefficients between a word’s phonological complexity based on the IPC and the WCM and its mean word accuracy across the different datasets (columns 2 and 3 of Table 3). Also included in the table is the mean production accuracy of the words in each database (column 6). The findings in columns 4 and 5 will be discussed later after the presentation of the statistical models. We observe that there was a strong correlation (.71 to .77) between a word’s phonological complexity based on the IPC and WCM and its accuracy in the datasets of the younger children, aged 2 to 3 years. That is, the more complexity points a word had, the less accurately the word was produced. Both word complexity indices were characterized by strong correlations. As for the datasets of the older children (Kehoe & Cretton, 2021; Kehoe & Girardier, 2020), the correlations were lower (.11 to .41) or nonsignificant, suggesting that a word’s phonological complexity only weakly related or did not relate to its production accuracy. The IPC seemed to capture word complexity effects best in dataset 3, whereas the opposite was true for dataset 4. It must be noted that the PCCs were very high in these databases, which may mean ceiling effects limited the influence of complexity on word production.

**Table 3.** Pearson correlation coefficients between a word’s phonological complexity based on the IPC, WCM, Custom, and Com4 indices and a word’s mean percent consonants correct (PCC) score.

Study	IPC^a^	WCM^b^	Custom^c^	Com4^d^	PCC
Kehoe and Havy (2019)	−.72***	−.71***	−.80***	−.72***	74.84
Kehoe et al. (2023)	−.71***	−.77***	−.76***	−.80 ***	72.78
Kehoe and Cretton (2021)	−.41*	−.32	−.62**	−.51**	93.42
Kehoe and Girardier (2020)	−.11	−.31**	−.67***	−.42***	93.58

a Complexity based on the Index of Phonetic Complexity (Jakielski, 2000, 2002).

b Complexity based on the Word Complexity Measure (Stoel-Gammon, 2010).

c Complexity index statistically derived for each dataset (this study).

d Complexity index based on four parameters (fricatives/liquids, word-final codas, clusters, alveopalatal fricatives, this study).

**Figure 1.**
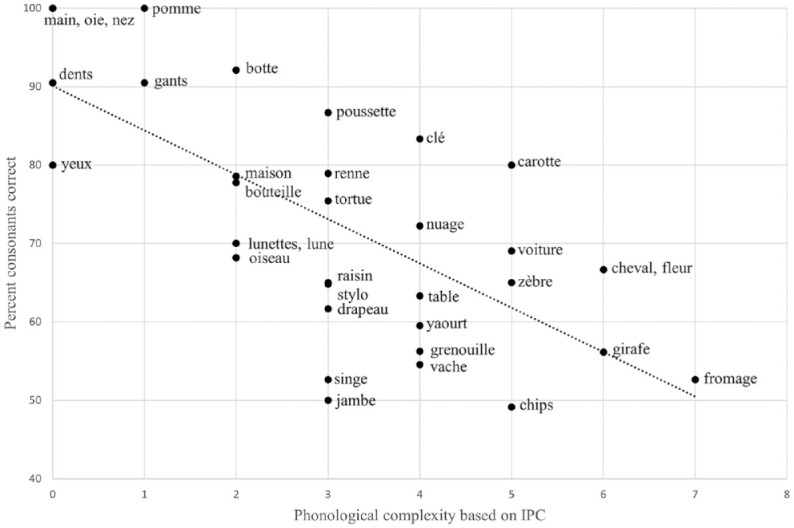
Scatterplot of the percent consonants correct for each word by their phonological complexity (as based on the IPC).

In sum, the correlation results indicated that a word’s phonological complexity based on the IPC and WCM negatively influenced its production accuracy, at least in children aged 2 to 3 years. As an illustration, we present an annotated scatterplot of the relation between IPC and PCC in the Kehoe et al. (2023) dataset (see Figure 1). Although there was a moderately strong relation between complexity and mean PCC per word, we can observe, at each complexity point, that there were certain words that received higher accuracy scores than others. For example, *lune*, *lunettes*, and *botte* have IPCs of 2. Yet, *botte* had higher word accuracy (92%) than *lune* and *lunettes* (70%). They all have word-final consonants; however, *lune* and *lunettes* have liquid /l/, whereas botte has PoA variegation of singleton consonants. In general, we observe that words containing fricatives, particularly alveopalatal fricatives (e.g., *jambe*, *singe*, *vache*, *chips*), had lower word accuracy than other words with the same number of complexity points.

### 3.2 Statistical models

According to the procedure outlined above, we ran several mixed-effects logistic regression models on all datasets to determine which factors best influenced consonant accuracy. In all cases, control (*age, gender, SES*) and predictor variables (phonological complexity, *PND, and Vocab*) were added to the models. The first and second models aimed to determine whether phonological complexity based on the *IPC* and *WCM*, respectively, influenced consonant accuracy; an interim model aimed to determine which individual complexity parameters influenced model fit; and the third model aimed to determine whether a new complexity index (*Custom*) was superior to that of the *IPC* and *WCM* in affecting consonant production.

#### 3.2.1 Dataset 1

The analyses conducted on dataset 1 involved 755 separate items for 17 children and 29 target words. Concerning control variables, *age* was not entered into the models as all children were the same age, that is, 2;6, and the control variables, *gender* and *SES*, did not emerge as significant in any of the models. The predictor variables, *Vocab* and *PND*, were significant in all of the models except for the Custom model, in which *PND* was only marginally significant. In models 1 and 2, both word complexity indices, the *IPC* and *WCM*, significantly influenced word accuracy. In the interim model, when individual complexity parameters were entered, four complexity parameters were significant (dorsals, fricatives/liquids, PoA variegation singletons, and clusters) and two parameters were marginally significant (word-final codas and alveopalatal fricatives). We calculated a new complexity index with the six factors taking into account the marginal effects. On the basis of AIC information criteria, the model containing the *Custom* index was superior to the one based on the *IPC* and the *WCM* (Custom index AIC: 1458; IPC AIC: 1464; WCM AIC: 1464). Thus, the factors that most influenced consonant accuracy in the Kehoe and Havy (2019) database were children’s vocabulary size and word complexity, the latter based on several different parameters: the presence of dorsals, fricatives/liquids, word-final consonants, clusters, PoA variegation of singletons, and alveopalatal fricatives. The results of models 1 (with *IPC*), 2 (with *WCM*), and 3 (with the *Custom* index) for dataset 1 are summarized in Appendix A.

#### 3.2.2 Dataset 2

The same procedure was repeated with the Kehoe et al. (2023) dataset for children aged 1;11 to 3;1. There were 695 separate items for 21 children and 35 target words. In none of the models, control variables were significant, whereas *Vocab* and *PND* improved model fit to data. In models 1 and 2, both the *IPC* and the *WCM* significantly influenced word production accuracy. In the interim model, two phonological complexity parameters were significant: fricatives/liquids and alveopalatal fricatives. We calculated a new complexity index based on these two parameters and entered it into the third model along with *PND* and *Vocab*. Based on the AIC information criteria, the Custom model was superior to the IPC model and slightly superior to the WCM model (Custom index AIC: 1121; IPC AIC: 1127; WCM AIC: 1123). Thus, increased consonant accuracy in the Kehoe et al. (2023) database was positively associated with superior vocabulary scores and words from denser phonological neighborhoods; it was negatively associated with a phonological complexity index that took into account the presence of fricatives/liquids and alveopalatal fricatives. Nevertheless, phonological complexity based on the *WCM* gave similar findings. The results of models 1, 2, and 3 for dataset 2 are summarized in Appendix B.

#### 3.2.3 Dataset 3

The models based on the Kehoe and Cretton (2021) dataset consisted of 1544 observations for 21 children and 25 words. The children were aged 2;7 to 4;3. In all models, the control variables, *sex* and *SES*, significantly influenced consonant accuracy along with *Vocab; age* and *PND* did not emerge as significant in any model. In the first model, *IPC* significantly influenced consonant accuracy, whereas, in the second model, the *WCM* only marginally influenced consonant accuracy. In the interim model, two parameters improved model fit to data: place variegation of clusters and the presence of alveopalatal fricatives. We calculated a new complexity index based on these two parameters and fitted a third model with the new complexity index. Results indicated that phonological complexity along with *gender, SES*, and *Vocab* all significantly accounted for consonant accuracy. The model based on the Custom index was superior to the one based on the IPC and the WCM (Custom index AIC: 1423; IPC AIC: 1432; WCM AIC: 1434). Thus, greater consonant accuracy in the Kehoe and Cretton (2021) dataset was associated with being a female, having higher SES, and knowing more words; it was negatively associated with producing words containing clusters with place variegation and alveopalatal fricatives. We also ran a model including the individual parameters, clusters (with or without place variegation), and alveopalatal fricatives. These two parameters together were significant but the variable, clusters with PoA variegation, along with alveopalatal fricatives, provided a superior model fit. The results of models 1, 2, and 3 for dataset 3 are summarized in Appendix C.

#### 3.2.4 Dataset 4

The models based on the Kehoe and Girardier (2020) dataset for children, aged 3;0 to 4;9, consisted of 1,797 individual items for 15 children and 85 words. In all models, *Vocab* influenced word production whereas *age, sex*, and *PND* did not. In this study, no measure of *SES* was collected. In the first model, the *IPC* marginally influenced consonant accuracy, whereas, in the second model, *WCM* significantly influenced consonant accuracy. In the interim model, three parameters—clusters, alveopalatal fricatives, and final voiced obstruents—were significant and one parameter, word-final consonants, was marginally significant. We calculated a new complexity index based on these four parameters, taking into consideration the marginal effect. Results indicated that the Custom index along with vocabulary knowledge was the best-fitting model. This model was superior to the one based on the IPC and WCM (Custom index AIC: 1361; IPC AIC: 1401; WCM AIC: 1388). Thus, in the Kehoe and Girardier (2020) dataset, consonant accuracy was positively related to knowing more words and negatively related to the production of words containing word-final consonants, clusters, alveopalatal fricatives, and final voiced obstruents. The results of models 1, 2, and 3 for dataset 4 are given in Appendix D.

### 3.3 Summary of statistical models and calculation of complexity index, Com4

To summarize, phonological complexity based on a reduced number of parameters (ranging from two to six parameters) was generally superior to phonological complexity based on the ICP or WCM in accounting for consonant accuracy across the four datasets. As can be seen in the fourth column of Table 3, the correlation coefficients between the mean PCC for each word and the Custom index were comparable (dataset 2) or higher (datasets 1, 3, and 4) than for the ICP and WCM. Production accuracy in dataset 1, consisting of children aged 2;6, was best accounted for by an index based on a range of parameters, many in common with the ICP (5 in common) and WCM (4 in common). Production accuracy in dataset 2, consisting of children aged 2 to 3 years, was best accounted for by an index of two parameters that resembled the WCM in terms of model fit. Thus, the IPC or WCM, although inferior to the Custom index in influencing consonant accuracy, fared reasonably well with the younger children’s productions. In the case of the older children’s productions, indices based on smaller numbers of parameters and different parameters (alveopalatal fricatives, final voiced obstruents) were superior to the IPC and WCM in accounting for children’s consonant accuracy. Table 4 summarizes the parameters that emerged as significant across the four datasets.

**Table 4.** Parameters that were implicated in the custom(-based) index of phonological complexity.

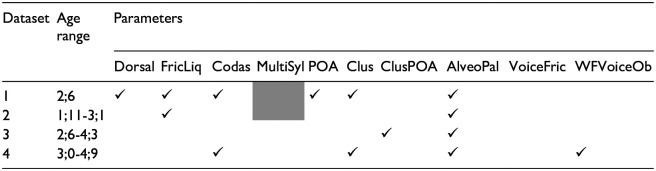

As a final analysis, we investigated whether a complexity index based on the parameters most frequently implicated in the Custom analyses (fricatives/liquids, word-final consonants, clusters, and alveopalatal fricatives) could serve as a reasonable approximation of complexity influences on consonant accuracy across the four databases. We refer to this index as Com4, a complexity index based on four parameters. We fitted new models with the four datasets entering the Com4 index along with vocabulary, PND, and any relevant control variables, as we had done with the other indices. The correlation coefficient between the mean PCC for each word and the Com4 index in the four datasets is listed in the fifth column of Table 3. The results of the mixed effects regression models using the Com4 index are shown as model 4 for all datasets in Appendices A to D. The correlation results indicate that the relation between the mean PCC score for each word and the Com4 index is significant for all datasets, the correlation coefficient being high for datasets 1 and 2 and moderate for datasets 3 and 4. In all regression models, phonological complexity based on Com4 significantly influenced production accuracy. On the basis of AIC scores, models using Com4 achieved comparable fit to the ICP/WCM (dataset 1) or to the Custom index (dataset 2) for the younger children, and were superior to the ICP/WCM but inferior to the Custom index for the older children (datasets 3 and 4). Thus, the Com4 could be considered as a compromise measure for examining complexity influences on consonant accuracy across the age range of 2 to 4 years.

### 3.4 Complexity analyses on production forms

**Figure 2.**
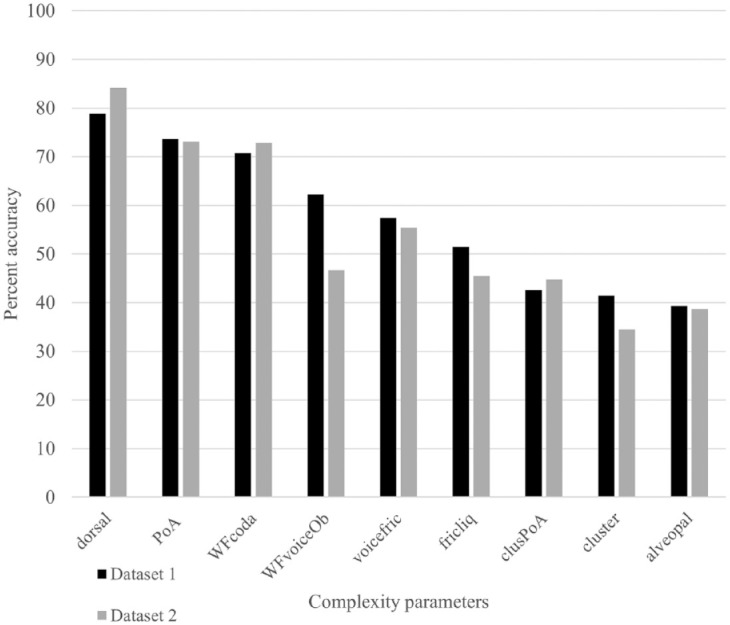
Percent correct accuracy of complexity parameters in children’s productions for datasets 1 (Kehoe & Havy, 2019; Children Aged 2;6) and 2 (Kehoe et al., 2023; Children Aged 1;11-3;1).

**Figure 3.**
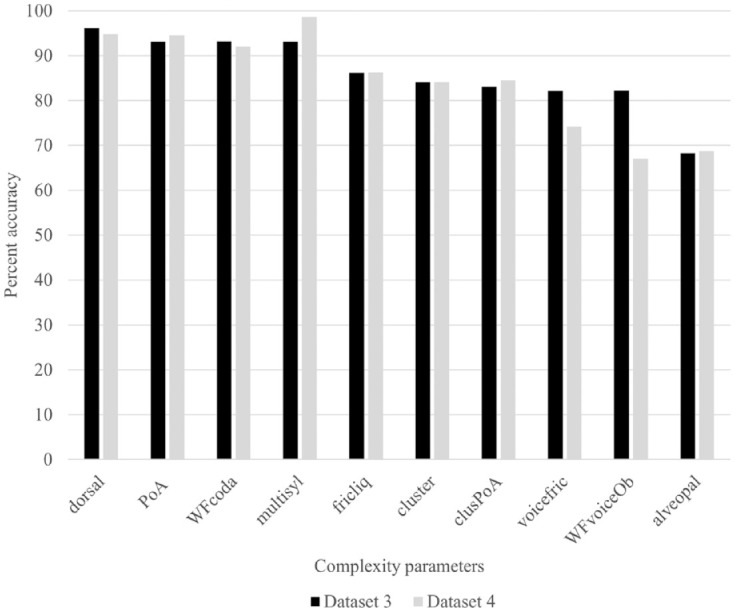
Percent correct accuracy of complexity parameters in children’s productions for datasets 3 (Kehoe & Cretton, 2021; Children Aged 2;7-4;3) and 4 (Kehoe & Girardier, 2020; Children Aged 3;0-4;9).

In additional analyses, we examined the accuracy of phonological sounds/structures in the children’s productions to confirm that complexity parameters in the target form were associated with poor accuracy in the output form. Figure 2 displays the findings for datasets 1 and 2 and Figure 3 displays the findings for datasets 3 and 4. These graphs reveal the percent accuracy of target-like realizations of each complexity parameter. For example, for word-final consonants, the graph shows the percentage of words in which target-like codas were produced. We considered target-like realizations and not simply the percentage of words in which codas (target-like or not) were produced because the measure PCC would have been sensitive to segmental accuracy (i.e., production of a nontarget-like coda would incur an error). The only exception was for the parameter, multisyllabic words, in which any production that matched the target word in terms of the number of syllables was considered correct. Finally, in words containing multiple items of one parameter (e.g., more than one fricative/liquid, cluster, alveopalatal fricative), only word productions in which all sounds/structures were target-like were considered correct.

If we examine the percent accuracy of complexity parameters in the youngest children’s productions (see Figure 2), we observe that dorsals, word-final consonants, and PoA variegation were characterized by high percent accuracy (greater than 70%) whereas fricatives/liquids, clusters, and alveopalatal fricatives were characterized by low percent accuracy (50% or lower). The results for both datasets 1 and 2 are similar except for final voiced obstruents, in which the children in dataset 2 do less well. The lower result may be because final voiced obstruents were less well sampled and consisted mainly of voiced alveopalatal fricatives. In the case of the older children’s productions (see Figure 3), all complexity parameters were characterized by high percent accuracy except for alveopalatal fricatives and final voiced obstruents (dataset 4), which received percent accuracy scores of less than 70%. Results were similar for both datasets except that children in dataset 4 appear to do less well with voiced fricatives and final voiced obstruents. In sum, the findings show that the complexity parameters are not characterized by equal difficulty. Certain parameters were not subject to error even in the younger children’s productions (dorsals, PoA variegation), and other parameters (alveopalatal fricatives) were subject to error in both the younger and older children’s productions. In general, the complexity parameters that emerged as significant in the Custom analyses were the parameters that were subject to the greatest numbers of errors.

### 3.5 Wholeword complexity effects on productions of clusters, codas, and alveopalatal fricatives

In the final analysis, we investigated if target sounds or structures were easier to produce if they were situated in less complex rather than in more complex words. If word complexity influences the accuracy of the target structure, we should see a reduction in the accuracy of the target structure with increasing word complexity. We focus on datasets 1 and 2 and the target structures, clusters, word-final codas, and alveopalatal fricatives because they were characterized by production variability. We used the IPC as the complexity index as it accounted for production accuracy reasonably well in datasets 1 and 2. In the case of clusters, we considered only word-initial C/l/ and C/r/ clusters because they were well-sampled in the data. We also analyzed them separately because studies show different acquisition rates for C/l/ and C/r/ clusters in French-speaking children (Kehoe et al., 2021). Table 5 presents the percent correct production of target C/l/, C/r/, word-final codas, and alveopalatal fricatives according to word complexity (ICP) in datasets 1 and 2.

**Table 5.** Percent correct production of C/l/ Clusters, C/r/ Clusters, word-final codas, and alveopalatal fricatives according to whole word complexity (Based on the IPC).

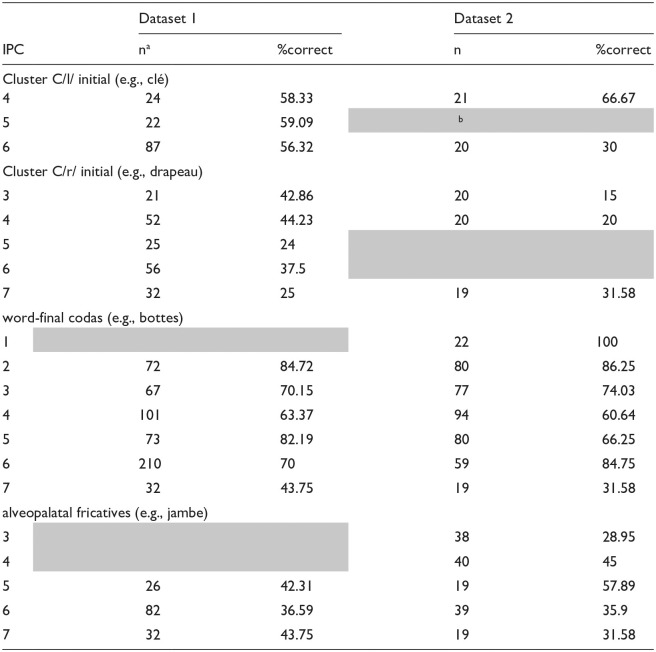

a n = number of words sampled across multiple children.

b Shaded areas indicate complexity levels that were not sampled.

A series of chi-square analyses were conducted to determine whether there were differences in percent accuracy according to complexity levels. Out of the eight chi-square analyses performed (four structures X two datasets), there were three analyses in which there were significant differences in percent accuracy according to complexity level. The production accuracy of word-final codas in datasets 1 and 2 varied according to complexity level, dataset 1: χ^2^(5) = 25.44, *p* < .001; dataset 2: χ^2^(6) = 44.95, *p* < .001. If we examine the results closely, however, we observe that the influence of complexity on production accuracy was not systematic. In both datasets, accuracy was high at low complexity levels, declining steadily until level 4 for dataset 1 and level 5 for dataset 2 before jumping to high accuracy and then declining again. It is true that accuracy was low at the highest complexity level (i.e., 7); however, this effect was mainly due to poor coda realization in the word *fromage* /fʁomaʒ/ “cheese,” which may relate to the difficulty of voiced alveopalatal fricatives as codas rather than word complexity effects per se. Production accuracy of C/l/ clusters in dataset 2 varied according to complexity level, χ^2^(1) = 5.51, *p* = .02,: clusters were realized more accurately in words with complexity level 4 versus 6. Once again, this may relate to cluster-type effects because these two complexity levels were characterized by two words, *cle* /kle/ “key” and *fleur* /flœʁ/ “flower,” and factors related to the respective difficulty of /kl/ versus /fl/ as clusters (due to sonority distance or manner of articulation of the first consonant of the cluster) may explain differences in accuracy rather than word complexity effects. In sum, there was not strong support for the influence of word complexity on the accuracy of a target sound or structure.

## 4 Discussion

Investigators have long recognized that having a simple index of a word’s phonological complexity is useful. Phonological complexity has been considered when constructing phonological tests (de Almeida et al., 2019; dos Santos & Ferré, 2018), when determining what factors influence vocabulary acquisition (Kehoe et al., 2023; Sosa & Stoel-Gammon, 2012), and when examining whether complex words are more prone to being dysfluent than noncomplex words (Dworzynski & Howell, 2004; Ratner, 2005). Few studies, however, have investigated the validity of complexity indices concerning production accuracy. This study asked whether words designated as complex based on word complexity indices are indeed more difficult to produce accurately than words designated as less complex. We coded the phonological complexity of words in four different databases of French-speaking children, aged 1;11 to 4;9, employing four different indices, two published measures, the IPC (Jakielski, 2000) and WCM (Stoel-Gammon, 2010), and two measures developed in this study, Custom and Com4, based on a smaller number of parameters and/or different parameters to the IPC and WCM. We investigated whether phonological complexity was related to production accuracy and determined which index best accounted for production accuracy in regression models that also included vocabulary knowledge and phonological neighborhood density. Additional analyses explored the realization of complexity features in the children’s productions and examined whether certain target structures (e.g., clusters, codas, alveopalatal fricatives) were produced more easily in words containing fewer rather than greater numbers of complexity features.

Findings revealed that all complexity indices accounted reasonably well for the youngest children’s productions; however, indices containing fewer and different parameters accounted best for the oldest children’s productions. A focus on children’s output forms revealed that not all complexity parameters posed difficulty: dorsal consonants and words containing place variegation were realized accurately even in the youngest children’s productions and alveopalatal fricatives were realized poorly even in the oldest children’s productions. We did not find any evidence for greater accuracy of target sounds and structures in low versus high complexity words. Thus, in terms of our main research aim, we observed that the IPC and WCM were valid measures of phonological complexity in relation to production accuracy when restricted to the age range of 2 to 3 years. They were no longer valid measures for the age range of 3 to 4 years. The phonological features that influenced production accuracy the most were fricatives/liquids, clusters, word-final codas, and alveopalatal fricatives. In the following paragraphs, we discuss the findings in greater detail focusing on the role of phonological complexity in production.

### 4.1 Summary of correlation analyses and statistical models

Datasets 1 and 2 consisted of children with a mean age of 2;6 and whose average PCC for word productions was 72–74%. We found that in all of the indices word complexity was highly correlated with the mean PCC per word (.71 to .80) and all significantly accounted for production accuracy in statistical models. This result is not surprising, given that the IPC and WCM were developed to capture the production difficulties of two-year-olds. Nevertheless, the IPC and WCM fell short of the mark in that not all complexity parameters served their purpose for analyzing these French data. In the Custom analyses, which entered all complexity parameters individually, certain complexity items did not emerge as significant or were significant in only one of the two datasets. If parsimony is a criterion, phonological complexity based on the Custom or Com4 indices would be preferable.

Datasets 3 and 4 consisted of children aged 3 to 4 years and whose mean PCC for word production was greater than 90%. We found that word complexity based on the IPC and WCM was only weakly (or not) correlated with the mean PCC per word and statistical models that included phonological complexity based on these indices had inferior model fit compared with those based on the Custom or Com4 indices. Clearly at ages 3 to 4, parameters such as the presence of dorsals, multisyllabic words, PoA variegation, and even fricatives/liquids no longer pose any production challenge to children. Their inclusion hinders rather than aids complexity measurement.

The Custom index achieved the best model fit in almost all datasets (it achieved comparable fit to Com4 in dataset 2), but this index cannot be used for practical purposes because its composition was derived from statistical manipulation and it varied greatly between datasets. The Com4 index was the next best index, achieving superior fit to the IPC and WCM in three of the datasets (datasets 2, 3, and 4) and comparable fit in one dataset (dataset 1). As for whether the IPC or WCM is superior, we did not observe a great difference between the measures. They achieved comparable model fit for dataset 1; the WCM was superior to the IPC for datasets 2 and 4, whereas the IPC was superior to the WCM for dataset 3.

We conducted the Custom analyses to determine which individual parameters influenced production accuracy the most. The presence of alveopalatal fricatives in the target word was the one parameter that influenced production accuracy across all datasets. In the children’s output forms, it was the parameter characterized by the lowest percent accuracy (see Figures 2 and 3). Several sources point to these as being late-acquired sounds, which are often targeted in phonological treatment (Aicart-de Falco & Vion, 1987; Kehoe & Philippart de Foy, 2023; MacLeod et al., 2011). They would have incurred multiple complexity points, being fricatives/liquids, sometimes voiced fricatives, and sometimes word-final consonants. They were, thus, weighted highly via the other parameters; however, the parameter itself *alveopalatal fricatives* still proved sensitive to production accuracy. Interestingly, the phonological parameter *voiced fricatives* (in the WCM only) did not emerge as significant in any of the custom-based analyses; other parameters having features in common with this parameter (fricatives/liquids, alveopalatal fricatives, final voiced obstruents) were more sensitive. The parameter *final voiced obstruents* emerged as significant in the dataset of the oldest group of children, consistent with several reports that this structure poses difficulty for children at the latest stages of phonological development (Glaspey et al., 2022; Kehoe, 2021). As suggested by the high percent accuracy of output forms in Figures 2 and 3, the parameters *dorsal* and *PoA variegation* were not associated with production difficulty but they still contributed to model fit in the dataset of the youngest children (dataset 1). Vihman et al. (2023) demonstrated that variegation (of both place and manner) creates production challenges for young children. The authors analyzed the data of children learning five different languages, including French, at the end of the single-word stage of development. The reduced influence of this parameter on production accuracy in the current study may be explained by the fact that the children were beyond the single-word period. *Word-final codas* were also realized with high percent accuracy by the youngest children; however, this parameter emerged as significant in two datasets (data sets 1 and 4). The parameter *fricatives/liquids* was a complexity parameter that posed difficulty for the youngest children. In terms of production accuracy, it was characterized by similar percent accuracy to *clusters*. In dataset 3, the parameter *clusters with PoA variegation* was a more sensitive indicator of production accuracy than *clusters* alone, whereas in two other datasets, the parameter *clusters* were the more sensitive measure. The parameter *multisyllabic words* did not emerge as significant in the Custom analyses. Multisyllabic words were also produced with high percent accuracy by children in datasets 3 and 4. We cannot determine if they would have posed a production challenge for the younger children because they were not sampled in these datasets. dos Santos and Ferré (2018) documented greater effects of complexity due to the presence of clusters than to increased word length on the accuracy of nonword repetition. Warnier et al. (2023) also documented ambiguous effects of word length: one-syllable words were characterized by poorer word accuracy than two- and three-syllable words.

The main age-related trend observed in the data was that the parameter *fricatives/liquids* emerged as significant in the regression analyses of the youngest children but no longer in those of the oldest children. The parameter *alveopalatal fricatives* and to a lesser extent *codas* and *clusters* influenced production accuracy at all ages. The correlational analyses also indicated that the influence of complexity on production accuracy was stronger in the younger than in the older age groups, as suggested by the greater magnitude of the correlation coefficients. The increased lexical knowledge in older children may play a role in the accurate production of challenging sounds and phonological structures; lexical knowledge was a significant factor influencing consonant accuracy in all age groups.

Apart from investigating the influence of word complexity on production accuracy, our regression analyses included other factors that may influence phonological production such as vocabulary, PND, and control factors. Across all datasets, the vocabulary level influenced production accuracy, consistent with many studies showing a close and possibly bi-directional relation between lexical and phonological development (Kehoe et al., 2015; Smith et al., 2006; Stoel-Gammon, 2011; Vihman, 2017). Our analyses also showed that PND influenced production accuracy. These findings are consistent with reports indicating that words from dense neighborhoods are produced more accurately than words from less dense neighborhoods (Kehoe et al., 2023; Sosa, 2008; Sosa, Stoel-Gammon, 2012). Saying words that have the same sound sequences as many other words facilitates word production. The effect was significant in the younger but not in the older children. This may be because PND effects on production accuracy decline with age: as children’s production skills improve, saying words with similar sound sequences no longer affords any advantage. The reduced effect may also be due to methodological factors because the data sets of the older children contained multisyllabic words, in which the effects of PND would be less pronounced (i.e., long words have fewer neighbors). Finally, the control factors, gender and SES, influenced production accuracy in one of the four datasets (dataset 3). Girls and children from higher SES had superior consonant accuracy. We have no explanation as to why these factors surfaced in only one dataset but, in general, the literature suggests that although gender and SES may influence speech sound production, it does not do so all the time (McLeod, 2009).

### 4.2 Crosslinguistic differences influencing complexity

Our study was conducted with French-speaking children whereas the IPC and WCM were developed for English-speaking children. Differences between the phonological features of French and English may explain why some of the complexity parameters were not sensitive to production accuracy in the current datasets. Compared with English, French has a higher proportion of multisyllabic words (MacLeod et al., 2011), a lower proportion of closed syllables (Delattre & Olsen, 1969), and a different rhythmic and stress system (Dell, 1984; Ramus et al., 1999). In terms of segmental differences, /r/ is realized as a uvular fricative in French and as an alveolar approximant in English; uvular /r/ is generally considered less marked and easier to acquire than alveolar /r/ (Kehoe, 2018; Ru͂ķe Draviņa, 1965; Yavas & Kehoe, 2024). The lateral /l/ in French, being light rather than dark (velarized), may also be acquired earlier in French- compared with English-speaking children (MacLeod et al., 2011; Yavas & Kehoe, 2024). Indeed, some studies suggest that French-speaking monolinguals obtain superior phonological scores to English-speaking monolinguals when compared with similar tasks. MacLeod et al. (2011) reported earlier mastery of certain consonants (e.g., /v, z, r/) in French-speaking preschool children aged 1;8–4;5 compared with English-speaking children of the same age. Brosseau-Lapré et al. (2018) also documented lower frequencies of phonological processes in typically developing French-speaking preschoolers compared with what is generally reported for English-speaking children. Other authors discuss the high accuracy of word-final codas in French (Kehoe, 2021) and the fact that final consonant deletion is not a frequent process in French phonologically disordered children, possibly because of the prosodic salience of word-final syllables (Brosseau-Lapré & Rvachew, 2014). Thus, segmental and prosodic differences between English and French may influence how complexity parameters affect production accuracy in the acquisition data.

Understanding the effects of phonological complexity on word production in different languages may help to illuminate the nature of phonological challenges in lexical development. Kehoe and colleagues show that word complexity based on the IPC is an important factor influencing vocabulary size in French-speaking children: children with larger vocabularies select words with greater phonological complexity (Kehoe et al., 2018, 2023). Stoel-Gammon (2011), in her review of the relationship between lexical and phonological development, points out that the variable rate of vocabulary acquisition across languages may, in part, be due to the phonological patterns of the target words. For example, Bleses et al. (2008) attribute the slow rate of vocabulary acquisition in Danish to the sound structure of the target words. Similarly, Tardif et al. (2009) cite phonology as one factor that may explain differences in vocabulary acquisition between Cantonese and Mandarin children: At the age of 2;0, Mandarin learners know more words than Cantonese learners (550 vs. 300). We assume that some of these effects relate to the phonological complexity of the target words in the respective languages.

### 4.3 Influence of word complexity on individual sounds and structures

Our analyses indicated that the more complexity parameters a word has, the less likely it will be produced with high accuracy. Our analyses did not show, however, that the more complexity parameters a word has, the less likely that a given structure will be produced with high accuracy. Some of the analyses showed that sound or structure accuracy varied according to word complexity level; however, on closer scrutiny, we could not determine whether such effects came from complexity features within the word or from the complexity of the individual structures per se. For example, the poor realization of the coda /ʒ/ in *fromage* could come about because it is a voiced alveopalatal fricative coda, that is, a late acquired coda, or from complexity effects elsewhere in the word such as the presence of a cluster. To address this effect more systematically, analyses would need to focus on a given target structure which would then be embedded progressively in more complex environments. We could analyze the accuracy of the /tr/ cluster, for example, in *train* /tʁɛ̃/ “train” (IPC = 3), *tram* /tʁam/ “tram” (4), *trésor* /tʁezɔːʁ/ “treasure” (6), and *tracteur* /tʁaktœːʁ/ “tractor” (7). If whole word complexity (complexity effects elsewhere in the word) influences the realization of /tr/, we should observe higher accuracy of the cluster in *train* than in *tracteur*. In the following section, we expand upon the implications of these findings in more detail.

### 4.4 Perspectives on complexity

So, what does this study tell us about phonological complexity? In the *Introduction*, we defined complexity in terms of structural considerations and articulatory difficulty (e.g., Lindblom & Maddieson, 1988). We found that two of the most sensitive complexity parameters related to syllable structure (word-final codas and clusters) and two related to articulatory difficulty (fricatives/liquids, alveopalatal fricatives), suggesting that both prosodic and segmental dimensions underlie complexity effects on production accuracy. This study examined two ways in which complexity influenced production accuracy. In the first set of analyses, we showed that complexity features were additive. The more complexity features a word had, the more likely it was to be produced with lower accuracy. Thus, the complexity of a word appears to be derived from the sum of the complexity of its individual structures/features, with the caveat that not all complexity parameters in current measures are equally difficult. In the second set of analyses, we examined whether producing a word that had a greater or smaller number of complexity parameters influenced the accuracy of a specific target structure. If the influence of complexity was simply additive then the accuracy of a target sound/structure would not necessarily be influenced by complexity features elsewhere in the word. Its accuracy would be dependent on the complexity of the individual sound/structure. Although the current findings were inconclusive, it is reasonable to assume that complexity is not just additive: producing multiple complex features/structures in a word may lead to interacting or cumulative effects that result in reduced accuracy of a target sound/structure. Such effects may be due to whole-word demands on phonological memory, phonological representation, and articulatory skills that limit resource allocation for individual sounds/structures.

Several examples in the literature suggest that target accuracy is influenced by complexity. In particular, studies document superior accuracy of sounds or structures in shorter versus longer words and in stressed versus unstressed syllables. Hilaire-Debove and Kehoe (2004), for example, found that word-final codas were realized more easily in one versus two-syllable words in French-speaking toddlers. Brosseau-Lapré and Rvachew (2014) reported higher reduction rates of word-initial clusters in two- and three-syllable words versus one-syllable words in phonologically disordered French-speaking children, aged four to five years. In the case of stress, Rose (2000) reported that two French-speaking two-year-olds realized clusters more accurately in stressed than in unstressed syllables. Bérubé et al. (2020) also found that sounds were more subject to deletion and substitution errors when situated in unstressed versus stressed syllables in French-speaking two- to four-year-olds. Other authors report that children may produce the same coda more accurately when it is situated in a high versus low phonotactic probability sequence in a nonword (e.g., gɛd vs. ʧʌd) (Zamuner et al., 2004), a finding that may also relate to complexity. Finally, Keren-Portnoy et al. (2010) found that a target sound was produced more accurately when it was embedded in a familiar word than in a non-word, evoking the importance of phonological memory for the whole-word sequence as playing a role. Thus, many isolated reports suggest that the accuracy of a given structure may be influenced by complexity-type effects occurring elsewhere in the word. It would be important to verify if complexity influences target accuracy when it is defined in terms of featural and structural parameters as in the indices studied in this research.

### 4.5 Limitations

The main limitation of this study is that it employed existing databases collected to test a wide range of phonological sounds and structures (codas, clusters, and alveopalatal fricatives) but not collected to test complexity effects alone. Future studies should employ databases that more systematically target words across a wide range of complexity levels. In particular, multisyllabic words were not sampled in the younger children so we cannot adequately address the influence of this complexity parameter. Percent accuracy was high in the older children, possibly resulting in ceiling effects that prevented complexity effects from surfacing. The inclusion of more complex words may lead to a stronger influence of complexity parameters on production accuracy than was observed in the current data. The data were cross-sectional and it would have been useful to study the influence of complexity in a longitudinal dataset to more carefully separate developmental from idiosyncratic child or word-related effects. To more precisely address whether whole word complexity influences target sound or structure accuracy, it would be necessary to select word stimuli so that the target structure is controlled, whereas the whole word environment is manipulated (e.g., *train*, *tram*, *tractor*).

## 5 Conclusion

Our study suggests that a useful measure for gauging the complexity of a word in French contains four parameters (fricatives/liquids, codas, clusters, and alveopalatal fricatives). Word complexity measures designed for English-speaking two-year-olds oversample complexity and are less sensitive to production accuracy in French than complexity indices containing fewer parameters. Across all age ranges, the presence of alveopalatal fricatives and to a lesser extent, the presence of codas and clusters predicted production accuracy, and, at the younger ages, the presence of fricatives/liquids did so as well. Future studies should address whether complexity based on the featural and structural properties in a word influences target sound and structure accuracy. Such studies would help to illuminate the nature of phonological complexity and how it influences the production of the whole word.

## Appendix

**Appendix A:** Models that examine the influence of phonological complexity on word production in dataset 1 (Kehoe & Havy, 2019).

Fixed effects	Estimate	Std. error	z value	Pr(>|*z*|)	
Model 1: IPC				AIC: 1464	BIC: 1492
(Intercept)	2.19669	0.25845	8.499	< 2e-16 ***	
Vocab	0.33066	0.15283	2.163	0.0305 *	
PND	0.18035	0.07872	2.291	0.0220 *	
IPC	-0.21564	0.04462	-4.832	1.35e-06 ***	
Random Effects					
Group Name	Variance	Std. Dev.			
Word (Intercept)	0.05632	0.2373			
Participant (Intercept)	0.36262	0.6022			
Model 2: WCM				AIC: 1464	BIC: 1491
(Intercept)	1.29129	0.16467	7.841	4.45e-15 ***	
Vocab	0.33209	0.15299	2.171	0.0300 *	
PND	0.20033	0.07901	2.536	0.0112 *	
WCM	-0.36959	0.07702	-4.799	1.60e-06 ***	
Random Effects					
Group Name	Variance	Std. Dev.			
Word (Intercept)	0.05953	0.2440			
Participant (Intercept)	0.36352	0.6029			
Model 3: Custom (dorsal + fricatives/liquids + clusters + word-final codas + place variegation singletons + alveopalatal fricatives)				AIC: 1458	BIC: 1486
(Intercept)	2.25485	0.23228	9.707	< 2e-16 ***	
Vocab	0.32862	0.15213	2.160	0.0308 *	
PND	0.12119	0.07013	1.728	0.0840	
New Index	-0.24094	0.03894	-6.187	6.14e-10 ***	
Random Effects					
Group Name	Variance	Std. Dev.			
Word (Intercept)	0.02197	0.1482			
Participant (Intercept)	0.35872	0.5989			
Model 4 : Com4				AIC: 1465	BIC: 1493
(Intercept)	1.97070	0.23025	8.559	< 2e-16 ***	
Vocab	0.33103	0.15286	2.166	0.0303 *	
PND	0.16146	0.08211	1.966	0.0493 *	
Com4	-0.22296	0.04882	-4.567	4.95e-06 ***	
Random Effects					
Group Name	Variance	Std. Dev.			
Word (Intercept)	0.06235	0.2497			
Participant (Intercept)	0.36276	0.6023			

* *p* < .05. ***p* < .01. ****p* < .001.

**Appendix B:** Models that examine the influence of phonological complexity on word production in dataset 2 (Kehoe et al., 2023).

Fixed effects	Estimate	Std. error	z value	Pr(>|*z*|)	
Model 1: IPC				AIC: 1127	BIC: 1154
(Intercept)	2.16235	0.31923	6.774	1.26e-11 ***	
Vocab	0.60380	0.15286	3.950	7.82e-05 ***	
PND	0.49078	0.15635	3.139	0.001696 **	
IPC	-0.26564	0.07669	-3.464	0.000533 ***	
Random Effects					
Group Name	Variance	Std. Dev.			
Word (Intercept)	0.3030	0.5504			
Participant (Intercept)	0.4492	0.6702			
Model 2: WCM				AIC: 1123	BIC: 1150
(Intercept)	1.3450	0.1889	7.121	1.07e-12 ***	
Vocab	0.6026	0.1528	3.944	8.01e-05 ***	
PND	0.4298	0.1468	2.928	0.00341 **	
WCM	-0.5582	0.1321	-4.226	2.38e-05 ***	
Random Effects					
Group Name	Variance	Std. Dev.			
Word (Intercept)	0.2392	0.4891			
Participant (Intercept)	0.4488	0.6699			
Model 3: Custom (fricatives/liquids + alveopalatal fricatives)				AIC: 1121	BIC: 1148
(Intercept)	1.91293	0.23605	8.104	5.33e-16 ***	
Vocab	0.60549	0.15292	3.959	7.51e-05 ***	
PND	0.47294	0.13613	3.474	0.000512 ***	
New Index	-0.42639	0.09341	-4.565	5.00e-06 ***	
Random Effects					
Group Name	Variance	Std. Dev.			
Word (Intercept)	0.2121	0.4605			
Participant (Intercept)	0.4498	0.6707			
Model 4 : Com4				AIC: 1119	BIC: 1147
(Intercept)	2.14860	0.26144	8.218	< 2e-16 ***	
Vocab	0.60604	0.15265	3.970	7.18e-05 ***	
PND	0.41940	0.13545	3.096	0.00196 **	
Com4	-0.34107	0.06991	-4.879	1.07e-06 ***	
Random Effects					
Group Name	Variance	Std. Dev.			
Word (Intercept)	0.1877	0.4333			
Participant (Intercept)	0.4479	0.6692			

* *p* < .05 ** *p* < .01 *** *p* < .001.

**Appendix C:** Models that examine the influence of phonological complexity on word production in dataset 3 (Kehoe & Cretton, 2021).

Fixed effects	Estimate	Std. error	z value	Pr(>|*z*|)	
Model 1: IPC				AIC: 1432	BIC: 1469
(Intercept)	3.56497	0.39599	9.003	< 2e-16 ***	
Gender	0.76741	0.31362	2.447	0.01441 *	
SES	-0.46622	0.16667	-2.797	0.00515 **	
Vocab	0.70800	0.16269	4.352	1.35e-05 ***	
IPC	0.14155	0.05476	-2.585	0.00974 **	
Random Effects					
Group Name	Variance	Std. Dev.			
Word (Intercept)	0.2686	0.5183			
Participant (Intercept)	0.3271	0.5719			
Model 2: WCM				AIC: 1434	BIC: 1472
(Intercept)	3.37291	0.40094	8.412	< 2e-16 ***	
Gender	0.76599	0.31366	2.442	0.01460 *	
SES	-0.46634	0.16669	-2.798	0.00515 **	
Vocab	0.70833	0.16272	4.353	1.34e-05 ***	
WCM	-0.13947	0.07414	-1.881	0.05994	
Random Effects					
Group Name	Variance	Std. Dev.			
Word (Intercept)	0.3139	0.5603			
Participant (Intercept)	0.3273	0.5721			
Model 3: Custom (clusters with POA variegation + alveopalatal fricatives)					
				AIC: 1423	BIC: 1460
(Intercept)	3.3022	0.2782	11.872	< 2e-16 ***	
Gender	0.7640	0.3125	2.445	0.0145 *	
SES	-0.4662	0.1661	-2.807	0.0050 **	
Vocab	0.7087	0.1621	4.374	1.22e-05 ***	
New Index	-0.6675	0.1485	-4.496	6.94e-06 ***	
Random Effects					
Group Name	Variance	Std. Dev.			
Word (Intercept)	0.1434	0.3787			
Participant (Intercept)	0.3238	0.5690			
Model 4 : Com4				AIC: 1428	BIC: 1465
(Intercept)	3.6427	0.3601	10.116	< 2e-16 ***	
Gender	0.7647	0.3129	2.444	0.014532 *	
SES	-0.4662	0.1663	-2.804	0.005053 **	
Vocab	0.7088	0.1623	4.367	1.26e-05 ***	
Com4	-0.2488	0.0728	-3.417	0.000632 ***	
Random Effects					
Group Name	Variance	Std. Dev.			
Word (Intercept)	0.2126	0.4611			
Participant (Intercept)	0.3251	0.5702			

* *p* < .05. ***p* < .01. ****p* < .001.

**Appendix D:** Models that examine the influence of phonological complexity on word production in dataset 4 (Kehoe & Girardier, 2020).

Fixed effects	Estimate	Std. error	z value	Pr(>|*z*|)	
Model 1: IPC				AIC: 1397	BIC: 1436
(Intercept)	3.8008	0.3960	9.597	< 2e-16 ***	
Vocab	0.5010	0.1969	2.545	0.0109 *	
IPC	-0.1166	0.0624	-1.871	0.0614	
Random Effects					
Group Name	Variance	Std. Dev.	Corr.		
Word (Intercept)	3.8287	1.9567			
IPC	0.0852	.2919	-0.99		
Participant (Intercept)	0.5980	0.7733			
Model 2: WCM				AIC: 1388	BIC: 1415
(Intercept)	3.4886	0.2434	14.335	< 2e-16 ***	
Vocab	.4957	0.1957	2.533	0.011300 *	
WCM	-0.4655	0.1206	-3.860	0.000114 ***	
Random Effects					
Group Name	Variance	Std. Dev.			
Word (Intercept)	0.642	0.8013			
Participant (Intercept)	0.590	0.7681			
Model 3: Custom (word-final codas + clusters + alveopalatal fricatives + voiced word-final obstruents)					
				AIC: 1361	BIC: 1388
(Intercept)	4.4519	0.2890	15.402	< 2e-16 ***	
Vocab	0.4858	0.1979	2.455	0.0141 *	
New Index	-0.7115	0.0953	-7.466	8.29e-14 ***	
Random Effects					
Group Name	Variance	Std. Dev.			
Word (Intercept)	0.2833	0.5323			
Participant (Intercept)	0.5982	0.7735			
Model 4 : Com4				AIC: 1381	BIC: 1409
(Intercept)	4.36946	0.32121	13.603	< 2e-16 ***	
Vocab	0.49566	0.19589	2.530	0.0114 *	
Com4	-0.35294	0.06898	-5.117	3.11e-07 ***	
Random Effects					
Group Name	Variance	Std. Dev.			
Word (Intercept)	0.5006	0.7075			
Participant (Intercept)	0.5927	0.7698			

* *p* < .05. ***p* < .01. ****p* < .001.

## Notes

1. The examiner was asked to elicit at least two productions of each stimulus word in the Kehoe and Havy (2019) and Kehoe and Girardier (2020) datasets. In the Kehoe and Cretton (2021) dataset, the examiner elicited three productions because this study focused also on intraword variability. Due to the young age of the children (1;11-3;1) and the number of target words (*n* = 35), only a single production of each word was elicited in the Kehoe et al. (2023) dataset.

2. The French /r/ is a voiced uvular fricative. In the past, we have coded it as having dorsal PoA (e.g., Kehoe et al., 2018). However, preliminary analyses of the production data indicated that it patterned very differently from /k, g/. Thus, we coded only dorsal stops as having dorsal PoA. Similarly, we did not assign complexity points to it for being a voiced fricative. Unlike other voiced fricatives (e.g., /v, z, ʒ/), it does not contrast for voicing. Furthermore, [ʁ], being present in many clusters and being also fricative/liquid, already receives multiple complexity points.

3. Stoel-Gammon (2010) defines clusters as a sequence of two or more consonants within a syllable; hence, we only assigned complexity points to tautosyllabic clusters.

4. Decisions about model selection were guided by criteria provided by Burnham and Anderson (2004). If a model was more than 2 AIC units lower than another, it was considered a superior model.
